# Older patient age and prior antimicrobial use strongly predict antimicrobial resistance in *Escherichia coli* isolates recovered from urinary tract infections among female outpatients

**DOI:** 10.1371/journal.pone.0285427

**Published:** 2023-05-11

**Authors:** Fanny S. Mitrani-Gold, Keith S. Kaye, Vikas Gupta, Aruni Mulgirigama, Barbara W. Trautner, Nicole E. Scangarella-Oman, Kalvin C. Yu, Gang Ye, Ashish V. Joshi

**Affiliations:** 1 GSK, Collegeville, Pennsylvania, United States of America; 2 Rutgers Robert Wood Johnson Medical School, New Brunswick, New Jersey, United States of America; 3 Becton, Dickinson and Company, Franklin Lakes, New Jersey, United States of America; 4 GSK, Brentford, Middlesex, United Kingdom; 5 Michael E. DeBakey Veterans Affairs Medical Center, Houston, Texas, United States of America; 6 Baylor College of Medicine, Houston, Texas, United States of America; University of Cape Coast, GHANA

## Abstract

**Background:**

Increasing prevalence of antimicrobial resistance (AMR), including multidrug resistance (MDR), among *Escherichia coli* (*E*. *coli*) makes treatment of uncomplicated urinary tract infection (uUTI) difficult. We assessed risk factors for fluoroquinolone (FQ)-not-susceptible (NS) and MDR *E*. *coli* among US female outpatients.

**Methods:**

This retrospective cohort study utilized data from female outpatients aged ≥ 12 years with *E*. *coli* positive urine culture and oral antimicrobial prescription ± 1 day from index. We assessed patient-level factors within 90 and 91–360 days prior to index as predictors of FQ NS (intermediate/resistant) and MDR (NS to ≥ 1 drug across ≥ 3 classes) *E*. *coli*: age, prior oral antimicrobial dispensing, prior AMR phenotypes, prior urine culture, and prior hospitalization.

**Results:**

Among 1,858 outpatients with urine-isolated *E*. *coli*, 369 (19.9%) had FQ NS and 59 (3.2%) had MDR isolates. After multivariable adjustment, independent risk factors (*p* < 0.03) for FQ NS *E*. *coli* were older age, prior FQ NS isolates, prior dispensing of FQ, and dispensing of any oral antibiotic. Independent risk factors (*p* < 0.02) for MDR were prior extended-spectrum β-lactamase-producing isolates (ESBL+), prior FQ dispensing, and prior oral antibiotic dispensing.

**Conclusions:**

In women with uUTI due to *E*. *coli*, prior dispensing of FQ or any oral antibiotic within 90 days predicted FQ NS and MDR urine *E*. *coli*. Prior urine culture with FQ NS isolates and older age were predictive of FQ NS *E*. *coli*. Prior ESBL+ was predictive of MDR *E*. *coli*. These data could help identify patients at risk for AMR *E*. *coli* and inform empiric prescribing.

## Introduction

Urinary tract infections (UTIs) are the most common outpatient bacterial infection in the US, with a lifetime risk of 50–60% for women [1]. Uncomplicated UTIs (uUTIs) occur in women without anatomical or functional abnormalities of the urinary tract, immunosuppression, pregnancy, or complicated diabetes. At least 10–12% of adult female patients in the US experience uUTIs each year, and 20–40% of these female patients have a subsequent recurrent uUTI infection [2, 3]. The majority of uUTIs are caused by *Escherichia coli* (*E*. *coli*) and treatment for uUTI is often empiric and without the use of urine culture or antibiotic susceptibility testing [4, 5]. However, antibiotic resistance has increased in the community setting and has spread to Enterobacterales species, including the most common urinary pathogens [6], bringing into question current practices for empiric treatment decisions. Patients at risk of infection caused by bacteria resistant to usual first-line therapies need to be identified prior to initiation of treatment to provide appropriate therapy and avoid unnecessary prolongation of symptoms and further courses of antibiotics. This study was conducted to examine real-world data from UTIs among US female outpatients with urine culture positive for *E*. *coli* treated with an antimicrobial, to determine risk factors for antimicrobial resistance (AMR).

## Materials and methods

### Study design and patients

This was a retrospective cohort study of data from women with outpatient UTI (positive urine culture and antimicrobial treatment within one day of culture) collected from nine outpatient facilities included in the BD Insights Research Database (Becton, Dickinson and Company, Franklin Lakes, New Jersey, US). This database has been described previously [7] and provides data from across all US census regions. Urine cultures were collected between January 1, 2015 and December 31, 2019. The primary objective was to identify patient-level risk factors or predictors of AMR (fluoroquinolone [FQ]-not-susceptible [NS] and multidrug resistance [MDR]) among *E*. *coli* isolates from adult and adolescent women with UTI. Data from female outpatients ≥ 12 years of age who had a urine culture positive for *E*. *coli* (index culture) collected in an outpatient setting (and not associated with inpatient admission within 24 hours), at least 12 months of baseline data, and 28 days of follow-up data available were included in the study. Patients were excluded if they were prescribed an intravenous (IV) or intramuscular antibiotic for the initial UTI (suggesting complicated UTI). Patients were also excluded if they had any of the following comorbidities at baseline: uncontrolled diabetes mellitus (hemoglobin A1C result > 8.0%), immunosuppression (prescription of immunosuppressive therapy), or pregnancy (prescription of oral folic acid and multivitamins). Outpatient UTI was defined as culture positive patients (≥ 10^4^ colony forming units/ml, based on Clinical and Laboratory Standards Institute [CLSI] standards) who received an oral antibiotic (nitrofurantoin [NFT]; trimethoprim-sulfamethoxazole [SXT]; fosfomycin; amoxicillin; amoxicillin-clavulanate; cefpodoxime; cephalexin; cefdinir; ciprofloxacin; or levofloxacin) ± 1 day of index culture collection. Eligible patients had *E*. *coli* isolates with facility-reported antimicrobial susceptibility results recorded in the electronic health record demonstrating: 1) susceptible or NS (resistant/intermediate) to FQ, or 2) MDR, defined as not susceptible to ≥ 1 drug across ≥ 3 drug classes (NFT, SXT, FQ, 3^rd^ or 4^th^ generation cephalosporin, or extended-spectrum β-lactamase production [ESBL+] via confirmatory panels), or no MDR. Susceptibility results were interpreted using US Food and Drug Administration and CLSI breakpoints and interpretive criteria [8]. Data collected from the 12-month baseline period included: age, geographic location, hospital characteristics (bed numbers, urban/rural status, teaching status), prior urine culture results, prior oral antibiotic prescription fill, prior AMR, and prior healthcare exposures (hospital admission with/without IV/oral antibiotics). The following predictors of AMR were evaluated up to 360 days prior to the index event: prior ESBL+, prior NS isolates (FQ, SXT, or NFT), prior urine culture (positive/negative), prior oral FQ, or other oral antibiotic (of those listed above) prescription fill. These predictors were stratified by timing of occurrence, within 90 days, and from 91 days to 360 days, prior to index. Other predictors examined were age, hospital size (≤ 300 or > 300 beds), hospital teaching status, and region (patients from East Central [41%], Middle Atlantic [25%], and West Central and Pacific [33%] regions of the US).

### Statistical analyses

The bivariate associations between AMR (FQ NS or MDR) *E*. *coli* isolates and each potential predictor were explored via univariate analysis using a Chi-square test or Fisher’s exact test as appropriate for categorical variables. In the multivariable modeling phase, generalized linear mixed models were used to examine the relationship between FQ NS (or MDR) and their respective predictor variables. The stepwise procedure was used as the first step of variable selection. The final model was selected based on statistical significance (*p* < 0.05) and goodness-of-fit statistics i.e., Akaike’s information criterion and Bayesian information criterion. Some effects that were deemed clinically important (such as prior FQ NS and prior ESBL+ *E*. *coli*), were included in the final model regardless of their significance (*p* values) if the bivariate assessment was statistically significant. Covariates for the modeling included patient age, baseline episode demographics, hospital characteristics (bed numbers [≤ 300 and > 300]; teaching status; urban/rural status), and geographic region. The effects of risk factors in the final model were assessed through odds ratios (ORs) with 95% confidence intervals (CIs) for each time period of occurrence.

### Ethical approvals

The study was conducted using a limited retrospective de-identified data set and exempt from consent by the New England Institutional Review Board/Human Subjects Research Committee (Wellesley, MA). The study was conducted in compliance with Health Insurance Portability and Accountability Act (HIPAA) requirements. The data used for this study complied with applicable laws regarding subject privacy, and there was no direct subject contact or primary collection of individual human subject data.

## Results

Overall, 1,858 women with outpatient UTI due to *E*. *coli* were included in the analysis. The mean age (standard deviation [range]) was 46.1 (22.8 [12–90]) years. At index, 369 (19.9%) patients had *E*. *coli* isolates that were FQ NS, and 59 (3.2%) had isolates that were MDR. Most patients with AMR *E*. *coli* (FQ or MDR) were in the 25–50 (39.0%) and > 50 (39.3%) year age groups. Using univariate analysis, only prior NFT NS isolates and hospital characteristics (bed size, teaching status, and region) did not reach the statistical threshold for inclusion in the final multivariate model of predictors for FQ NS or MDR *E*. *coli* isolates (S1 Table).

### Age

**Fig 1:**
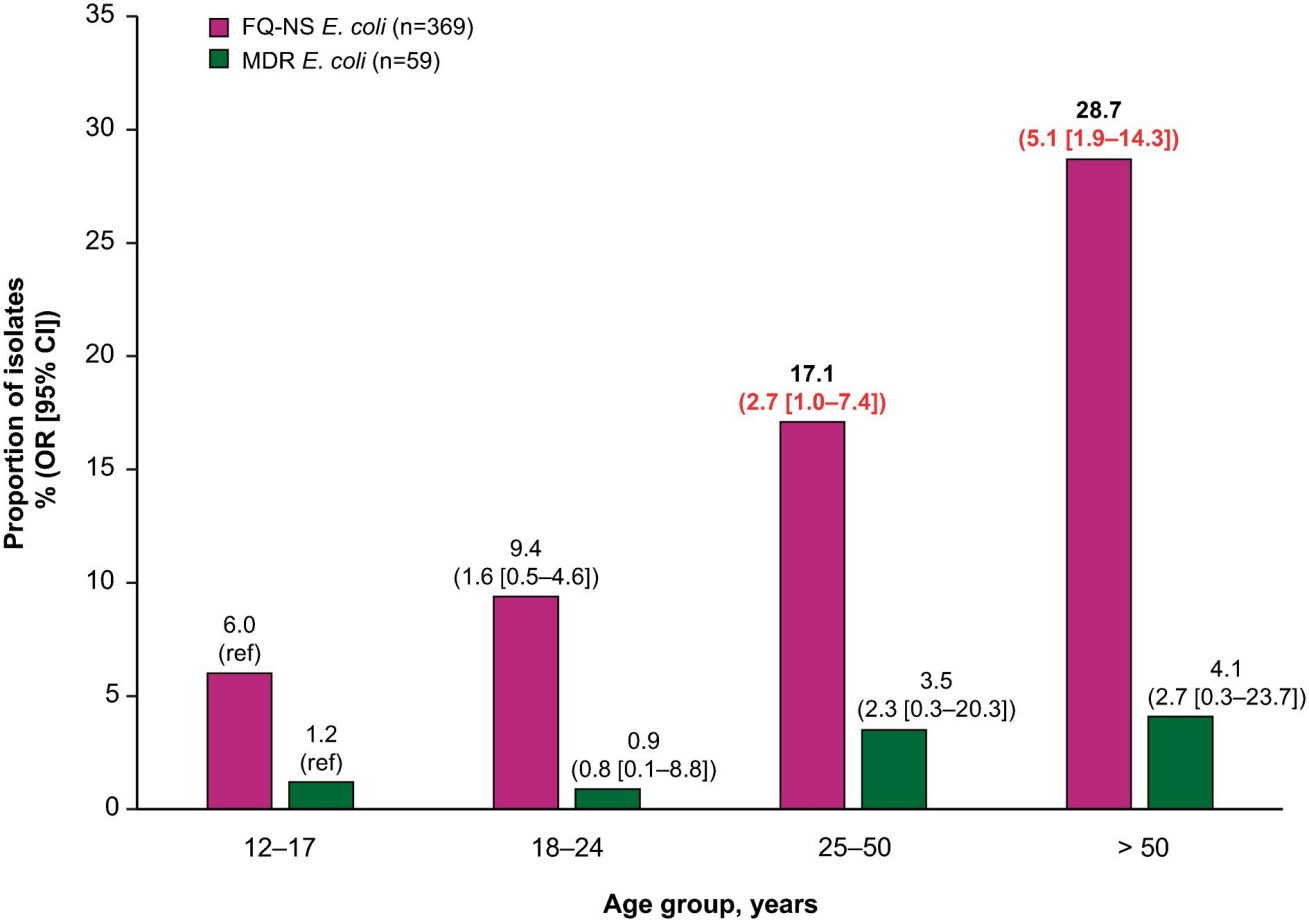
Association between patient age and risk of FQ NS or MDR *E*. *coli* isolates from urine*. Note: proportions from univariate analysis, OR (95% CI) from multivariate analysis. Red indicates *p* < 0.05. *Multivariate analysis utilized general linear mixed models with the following variables: prior ESBL+, prior NS isolates (FQ, SXT, or NFT), prior urine culture (positive/negative), prior oral FQ or other oral antibiotic prescription fill, age, hospital size (≤ 300 or > 300 beds), hospital teaching status, and region. CI, confidence interval; *E*. *coli*, Escherichia coli; ESBL+ extended-spectrum β-lactamase producing; FQ fluoroquinolone; MDR multidrug-resistant; NFT nitrofurantoin; NS not susceptible; OR odds ratio; ref reference; SXT trimethoprim-sulfamethoxazole.

After multivariable adjustment (Table 1), age was found to be an independent risk factor (*p* < 0.0001) for patients having FQ NS *E*. *coli* isolates at index (Fig 1). The youngest age group (12–17 years) was used as the reference and a stepwise association between older age and risk of FQ NS patients with *E*. *coli* was found, with an OR of 2.7 (95% CI: 1.0–7.4) at 25–50 years, and 5.1 (95% CI: 1.9–14.3) at > 50 years (Fig 1). A larger proportion of patients aged 25–50 and > 50 years had MDR *E*. *coli* isolates compared to younger patients, but no independent association was found between age and risk of MDR among patients with *E*. *coli* isolates at index (Fig 1).

**Table 1:** Multivariable adjusted analysis for risk factors of FQ NS and MDR among *E*. *coli* isolated from the urine of US women 2015–2019*.

Factors	FQ NS (N = 369)				MDR (N = 59)			
	n	%	OR (95% CI)	*P*	n	%	OR (95% CI)	*P*
**Age group, years**				**< 0.0001**				0.2576
12–17	5	6.0	reference		1	1.2	reference	
18–24	30	9.4	1.6 (0.5–4.6)		3	0.9	0.8 (0.1–8.8)	
25–50	124	17.1	2.7 (1.0–7.4)		25	3.5	2.3 (0.3–20.3)	
> 50	210	28.7	5.1 (1.9–14.3)		30	4.1	2.7 (0.3–23.7)	
**Prior ESBL+**				0.7246				**0.0013**
≤ 90 days prior to index	17	73.9	1.3 (0.3–5.2)		9	39.1	8.4 (2.1–33.9)	
91–360 days prior to index	10	45.5	0.7 (0.2–2.1)		4	18.2	6.8 (1.4–32.7)	
No prior AMR	342	18.9	reference		46	2.5	reference	
**Prior FQ NS**				**< 0.0001**				0.4275
≤ 90 days prior to index	50	90.9	36.4 (13.1–100.9)		12	21.8	2.0 (0.7–6.0)	
91–360 days prior to index	44	63.8	9.1 (4.9–16.8)		4	5.8	0.8 (0.2–3.1)	
No prior AMR	275	15.9	reference		43	2.5	reference	
**Prior oral FQ receipt (any)**				**< 0.0001**				**0.0045**
≤ 90 days prior to index	68	54.0	3.3 (2.0–5.4)		15	11.9	3.2 (1.4–7.5)	
91–360 days prior to index	72	33.5	1.6 (1.1–2.5)		15	7.0	3.3 (1.5–7.4)	
No prior AMR	229	15.1	reference		29	1.9	reference	
**Prior oral AB receipt (any)**				**0.0284**				**0.0199**
≤ 90 days prior to index	168	32.6	**1.6 (1.1–2.3)**		37	7.2	**3.0 (1.2–7.3)**	
91–360 days prior to index	94	21.0	1.2 (0.8–1.8)		12	2.7	1.3 (0.5–3.4)	
No prior AMR	107	12.0	reference		10	1.1	reference	
**Hospital bed size**				0.001				0.0009
≤ 300	39	15.4	reference		3	1.2	reference	
> 300	330	20.6	2.8 (1.7–4.5)		56	3.5	10.8 (2.7–43.3)	
**Hospital teaching status**				0.0380				
Non-teaching	235	21.2	1.4 (1.0–1.8)		38	3.4	Not included in final model	
Teaching	134	17.9	Reference		21	2.8		
**Region**			Not significant					0.0311
East Central	163	21.2			20	2.6	0.8 (0.4–1.6)	
Middle Atlantic	94	20.0			21	4.5	2.2 (1.0–5.0)	
West Central & Pacific	112	18.1			18	2.9	reference	

*Two variables, “Prior AMR-ESBL+” in FQ model and “Prior AMR-FQ” in MDR model, were included in the final models regardless of their statistical significance (*p* values) as they are deemed to be important clinical factors of FQ and MDR.

AB, antibiotic; AMR, antimicrobial resistance; CI, confidence interval; *E*. *coli*, *Escherichia coli*; ESBL+, extended-spectrum beta lactamase producing; FQ, fluoroquinolone; MDR, multidrug-resistant; NS, not susceptible; OR, odds ratio.

### Prior AMR phenotypes

**Fig 2:**
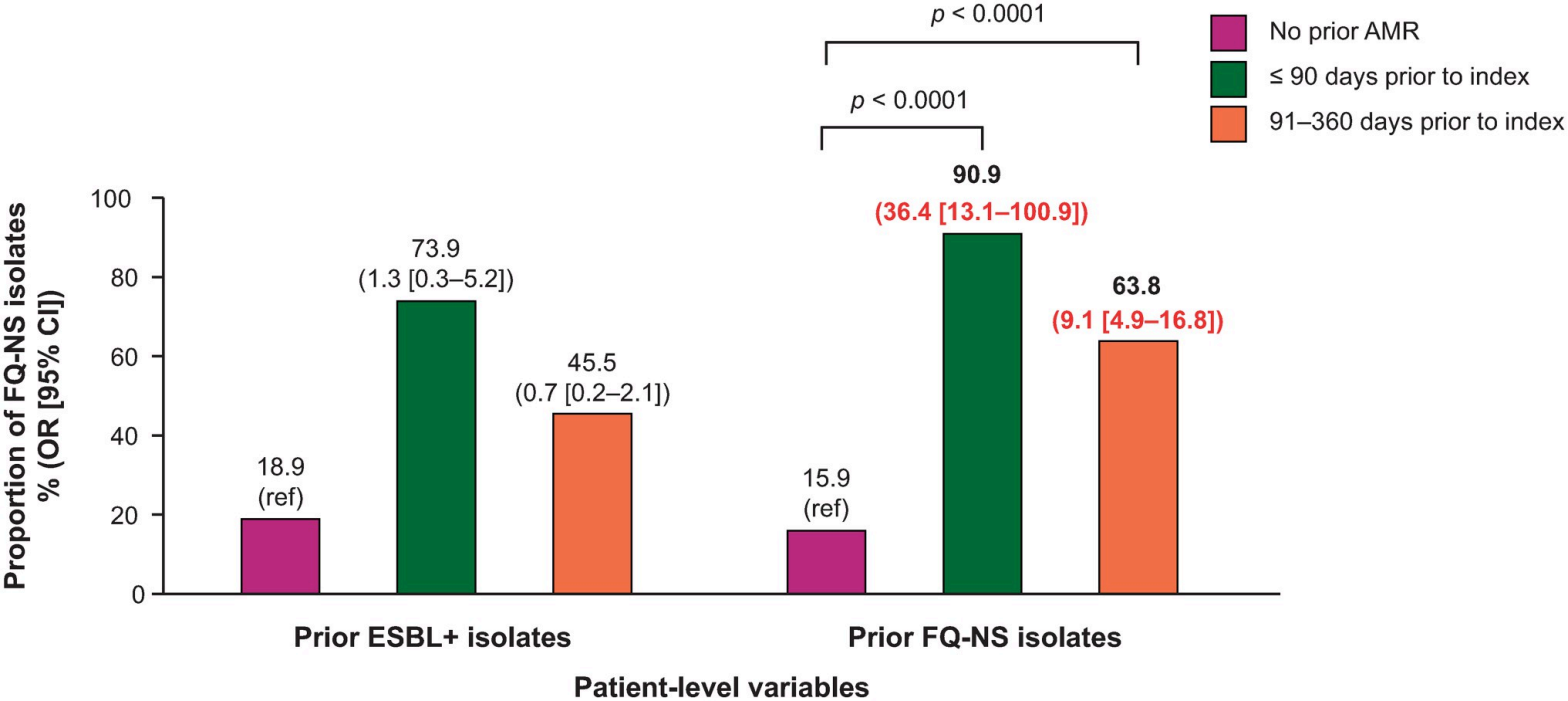
The association between prior antimicrobial resistance phenotypes and risk of FQ NS *E*. *coli* isolates at index*. Note: proportions from univariate analysis, OR (95% CI) from multivariate analysis. Red indicates *p* < 0.05. *Multivariate analysis utilized general linear mixed models with the following variables: prior ESBL+, prior NS isolates (FQ, SXT, or NFT), prior urine culture (positive/negative), prior oral FQ or other oral antibiotic prescription fill, age, hospital size (≤ 300 or > 300 beds), hospital teaching status, and region. AMR, antimicrobial resistance; CI, confidence interval; *E*. *coli*, Escherichia coli; ESBL+, extended-spectrum β-lactamase producing; FQ, fluoroquinolone; NFT, nitrofurantoin; NS, not susceptible; OR, odds ratio; ref, reference; SXT, trimethoprim-sulfamethoxazole.

Prior FQ NS urinary isolates in patients during baseline (confirmed with susceptibility test results), independently predicted the presence of FQ NS *E*. *coli* isolates at index. This association was present for patients with prior FQ NS isolates identified within 90 days (OR = 36.4; 95% CI: 13.1–100.9; *p* < 0.0001) and 91–360 days (OR = 9.1; 95% CI: 4.9–16.8; *p* < 0.0001) prior to index (Fig 2).

**Fig 3:**
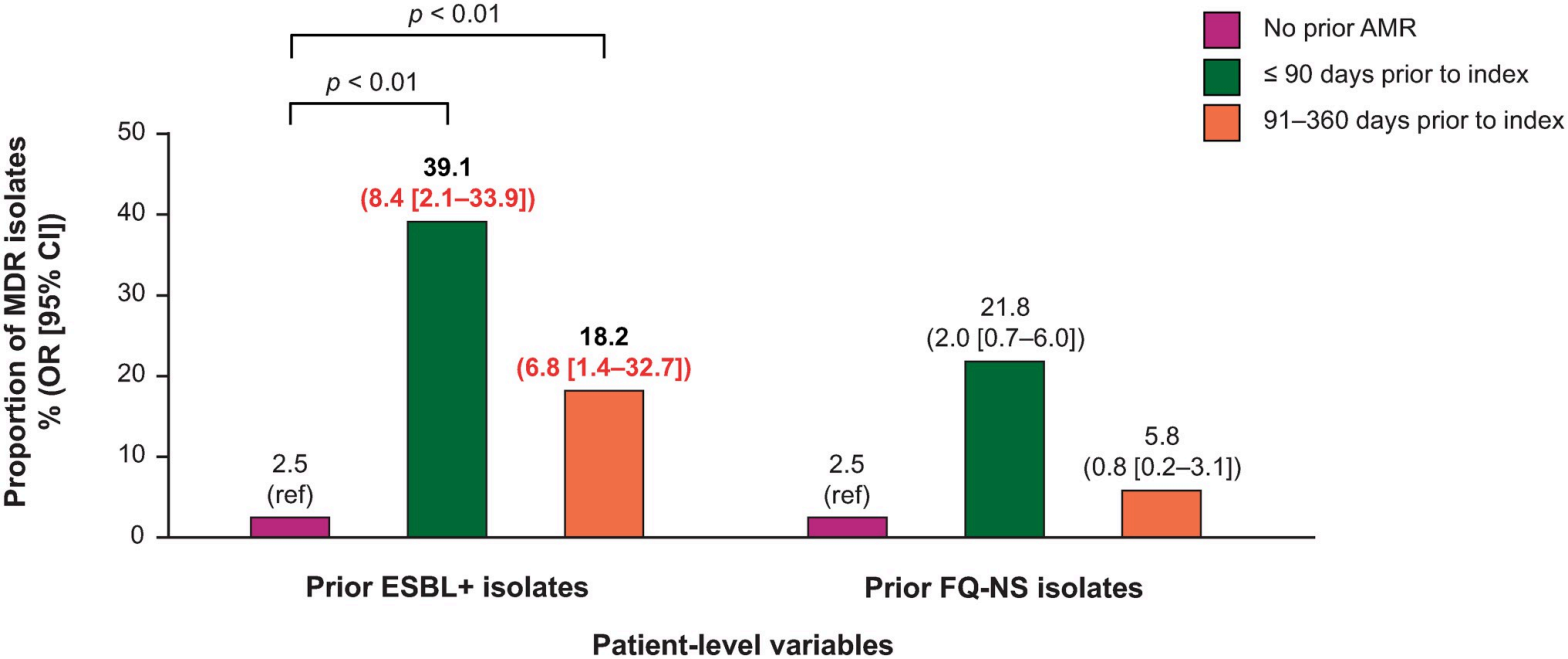
The association between prior resistance phenotypes and risk of MDR *E*. *coli* isolates at index*. Note: proportions from univariate analysis, OR (95% CI) from multivariate analysis. Red indicates *p* < 0.05. *Multivariate analysis utilized general linear mixed models with the following variables: prior ESBL+, prior NS isolates (FQ, SXT, or NFT), prior urine culture (positive/negative), prior oral FQ or other oral antibiotic prescription fill, age, hospital size (≤ 300 or > 300 beds), hospital teaching status, and region. AMR, antimicrobial resistance; CI, confidence interval; *E*. *coli*, Escherichia coli; ESBL+, extended-spectrum β-lactamase producing; FQ, fluoroquinolone; MDR, multidrug-resistant; NFT, nitrofurantoin; NS, not susceptible; OR, odds ratio; ref, reference; SXT, trimethoprim-sulfamethoxazole.

Prior resistance (ESBL+) was included as a covariate in the final model; however, this was not a predictor of FQ NS *E*. *coli* isolates at index (Fig 2). Having had a prior ESBL+ urinary isolate was a predictor of MDR *E*. *coli* at index, when identified in the 90 days prior to index uUTI (OR = 8.4; 95% CI: 2.1–33.9; *p* < 0.01), or from 91–360 days prior to the index uUTI (OR = 6.8; 95% CI: 1.4–32.7; *p* < 0.01) (Fig 3). Prior FQ NS isolates were not predictive of MDR *E*. *coli* isolates at index (Fig 3).

### Prior oral antibiotic prescription fill

**Fig 4:**
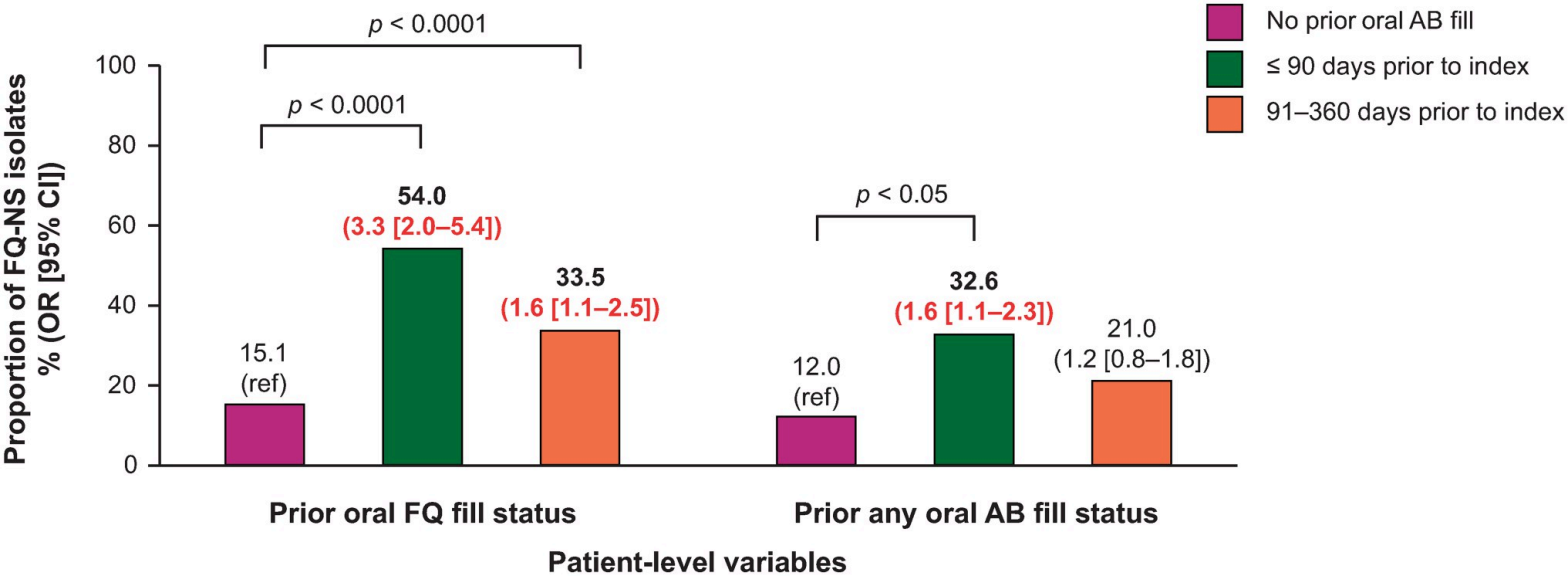
The association between prior antibiotic prescription fill and risk of FQ NS *E*. *coli* isolates at index*. Note: proportions from univariate analysis, OR (95% CI) from multivariate analysis. Red indicates *p* < 0.05. *Multivariate analysis utilized general linear mixed models with the following variables: prior ESBL+, prior NS isolates (FQ, SXT, or NFT), prior urine culture (positive/negative), prior oral FQ or other oral antibiotic prescription fill, age, hospital size (≤ 300 or > 300 beds), hospital teaching status, and region. AB, antibiotic; CI, confidence interval; *E*. *coli*, Escherichia coli; ESBL+, extended-spectrum β-lactamase producing; FQ, fluoroquinolone; NFT, nitrofurantoin; NS, not susceptible; OR, odds ratio; ref, reference; SXT, trimethoprim-sulfamethoxazole.

**Fig 5:**
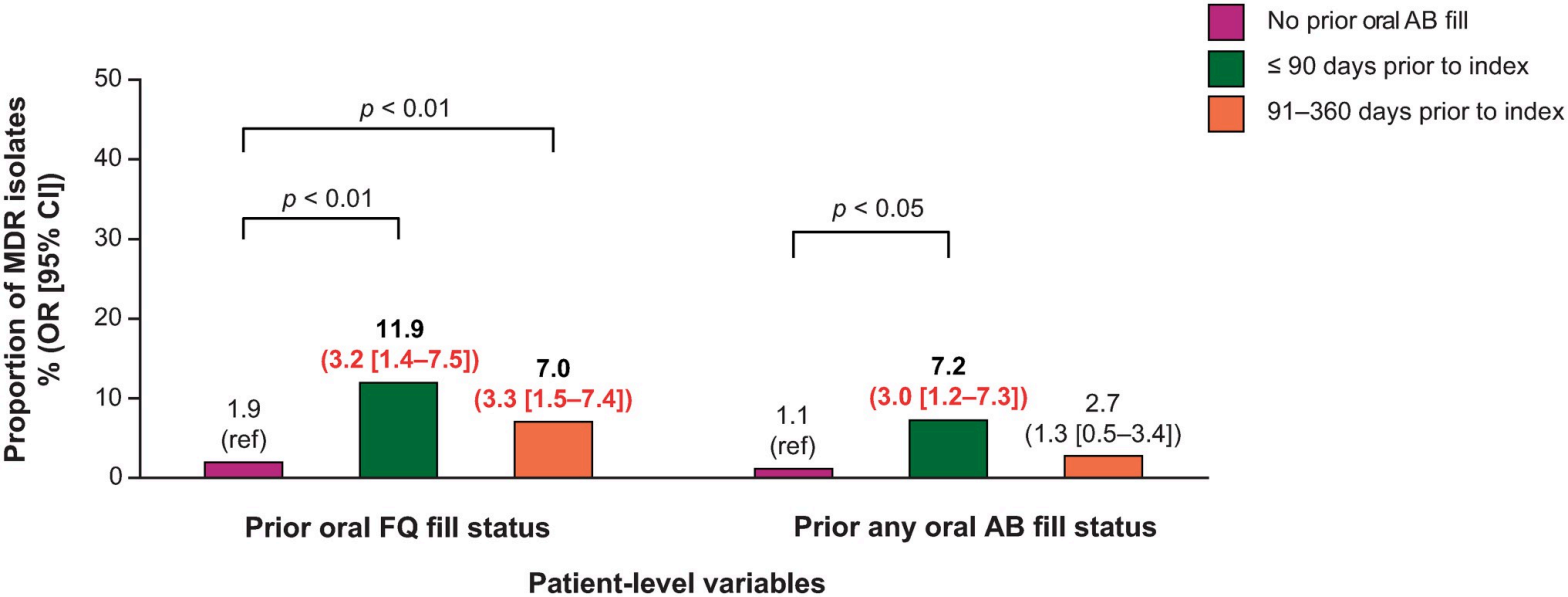
The association between prior antibiotic prescription fill and risk of MDR *E*. *coli* isolates at index*. Note: proportions from univariate analysis, OR (95% CI) from multivariate analysis. Red indicates *p* < 0.05. *Multivariate analysis utilized general linear mixed models with the following variables: prior ESBL+, prior NS isolates (FQ, SXT, or NFT), prior urine culture (positive/negative), prior oral FQ or other oral antibiotic prescription fill, age, hospital size (≤ 300 or > 300 beds), hospital teaching status, and region. AB antibiotic; CI, confidence interval; *E*. *coli*, Escherichia coli; ESBL+, extended-spectrum β-lactamase producing; FQ, fluoroquinolone; MDR, multidrug-resistant; NFT, nitrofurantoin; NS, not susceptible; OR, odds ratio; ref, reference; SXT, trimethoprim-sulfamethoxazole.

Prior prescription fill of oral FQ was a predictor of patients having an FQ NS *E*. *coli* isolate at index uUTI (Fig 4). Prescription fill of oral FQ within 90 days (OR = 3.3; 95% CI: 2.0–5.4; *p* < 0.0001) and 91–360 days (OR = 1.6; 95% CI: 1.1–2.5; *p* < 0.0001) prior to index was also independently predictive of FQ NS *E*. *coli* at index. The influence of prior oral FQ prescription fill was noticeably higher if prescribed within 90 days prior to index. Prior fill of any oral antibiotic prescription (including FQ) was also found to be a predictor of FQ NS among patients with index *E*. *coli* isolates, if prescription was filled within 90 days prior to index uUTI (OR = 1.6; 95% CI: 1.1–2.3; *p* < 0.05) (Fig 4). Prior fill of oral FQ prescription was a predictor of patients having MDR *E*. *coli* isolates when dispensed within 90 days prior to index (OR = 3.2; 95% CI: 1.4–7.5; *p* < 0.01), or from 91–360 days prior to index (OR = 3.3; 95% CI: 1.5–7.4; *p* < 0.01); fill of any prior oral antibiotic prescription was a predictor of patients having MDR *E*. *coli* isolates when dispensed within 90 days prior to index (OR = 3.0; 95% CI: 1.2–7.3; *p* < 0.05) (Fig 5).

## Discussion

Over the last 20 years, there has been a noticeable increase in AMR among *E*. *coli* isolates from community acquired uUTIs [6, 9]. In 2012, a study of *E*. *coli* isolates from US outpatients showed that there were significant increases in ciprofloxacin and SXT resistance between 2001 and 2010 [9]. Similarly, a study of *E*. *coli* urine isolates from 18 European countries found resistance to SXT and FQ in 33% and > 20% of samples, respectively [10]. Previously, we investigated *E*. *coli* isolates collected from urine of adolescent and adult female outpatients in the US between 2011 and 2019 for rates of FQ NS, NFT NS, SXT NS, and ESBL+ [6]. Among more than 1.5 million *E*. *coli* isolates, we found that rates of MDR *E*. *coli* had an average yearly increase of 2.7%, and that 21.1% of *E*. *coli* isolates collected in this period were NS to FQ [6].

AMR limits the available treatment options for empiric therapy by increasing the likelihood of treatment failure. This increases the burden on patients and healthcare systems, and leads to greater exposure to multiple classes of antibiotics, thereby potentially exacerbating drug resistance [11]. In the current study, patient-level real-world data were analyzed to determine risk factors for FQ NS or MDR among *E*. *coli* isolates from uUTI. Predictor variables were assessed in 2 time periods–within 90 days, and from 91–360 days prior to index. Age had a stepwise association with risk of FQ NS among outpatients with UTI and *E*. *coli*. Risk of index FQ NS *E*. *coli* isolates increased from the age of 25 years relative to age 12–17 years. Lifetime antibiotic exposure increases with age, which may explain the higher prevalence of resistant isolates at older ages. A systematic review of resistance to FQ in *E*. *coli* from community-acquired uUTIs did not find a consistent relationship between age and resistance [12]; however, several of the studies reviewed agreed with our conclusions [13–15]. Two studies found that FQ resistance was higher among postmenopausal women when compared to premenopausal women [16, 17], and another study found an association between FQ resistance and uUTI recurrence, which is more common among older women [18]. The differences observed between the studies included in the systematic review by Stapleton et al. and the current study may relate to differing methodologies, which may also explain the lack of consistency described by Stapleton et al.

Interestingly, age was not a significant predictor of patients having MDR *E*. *coli* isolates in this study, but we did see a trend towards increased MDR with age, and the lack of statistical significance could be due to the small sample size of the MDR group. A recent study by Kourtis et al. evaluating device and procedure-related infections (including catheter-associated UTIs), determined that while there was an association between being > 17 years of age and the likelihood of having MDR *E*. *coli*, this association did not increase with older age [19].

We found that both prior FQ NS and prior oral FQ prescription fill independently predicted patients having FQ NS *E*. *coli* isolates at index. Also, prior fill of FQ prescription and having prior urinary ESBL+ isolates were independent predictors of index MDR *E*. *coli* when occurring within 90 days and, importantly, 91–360 days before index. Associations were stronger when the predictors or risk factors occurred in the 90 days before index; however, these results demonstrate the longevity of influence that bladder infection with AMR organisms can have on future infections. These results also agree with a 2022 study which combined whole-genome sequencing of 1,113 pre- and post-treatment bacterial isolates with machine-learning analysis of 140,349 urinary tract infections and 7,365 wound infections [20]. The study found that emergence of resistance was common and driven by rapid reinfection with a different strain resistant to the prescribed antibiotic rather than by de novo evolution of resistance. The authors suggested that resistance-gaining recurrences could be predicted at the patient level using past infection history and minimized by machine learning-personalized antibiotic recommendations, offering a means to reduce the emergence and spread of resistant pathogens.

A further finding was that prior fill of any oral antibiotic prescription (for any etiology) within 90 days before index was a risk factor for patients having FQ NS or MDR *E*. *coli* isolates. This finding is consistent with the report by Stewardson et al. who found that FQ exposure transiently suppressed intestinal Enterobacterales and increased the probability of colonization with FQ-resistant strains [21].

Study limitations include the fact that the data in the study were sourced from only 9 facilities in the US, limiting the generalizability of the results; however, the data were taken from 3 census regions across the US. Given that outpatient UTI is often treated empirically without urine culture procurement, the inclusion of only patients with a urine culture result may have predisposed the study population towards inclusion of a higher proportion of recurrent uUTI (for which urine culture is recommended), causing potential bias towards higher AMR rates. The inclusion of patients with urine culture results was necessary to study predictors of resistance. We did not cover the entire spectrum of bacteria causing outpatient UTI because we limited our study to *E*. *coli*, the predominant pathogen causing outpatient UTI. We excluded male patients and women with complicated diabetes, severe renal impairment immunosuppression, and pregnancy based on laboratory results or medication fills, to facilitate removal of probable complicated UTI from this study population; however, these data did not permit exclusions based on the presence of all urological abnormalities or procedures associated with complicated UTI. Any potential misclassification of the study population would be non-differential and would have biased results towards the null. Furthermore, certain AMR risk factors identified by other studies were not evaluated because these data were not available. Finally, our data on antibiotic prescription fills do not indicate if the therapy was actually taken as prescribed.

Future research evaluating the effects of better-informed empirical therapy for outpatient UTI may demonstrate the clinical applications of our findings. More targeted initial therapy predicated on past antibiotic exposure, patient demographics, and resistance profiles may help improve successful initial therapy and deter cultivation of drug resistance. Finally, other ramifications of prolonged antimicrobial use and/or increased exposure to multiple classes of antibiotic—such as adverse drug events and *Clostridioides difficile* infection—are also relevant to our findings, as more appropriate antimicrobial prescribing could mitigate some of the risk factors for those downstream clinical consequences [20–24].

## Conclusions

The results described here highlight the need for careful consideration of patient history in the empiric treatment of uUTI, most notably their recent history of antibiotic prescription(s) across all indications and isolate susceptibility results. Risk factors occurring up to 12 months prior to index urine culture must also be considered. Our data are intended to be used to help identify patients with uUTI who are potentially at risk for AMR *E*. *coli*, and to inform effective empiric prescribing for uUTI.

## Supporting information

S1 Table Univariate analysis for risk factors of FQ NS and MDR *E*. *coli* isolated from urine of US women 2015–2019. (DOCX)
