# Supplementary material for: Older patient age and prior antimicrobial use strongly predict antimicrobial resistance in Escherichia coli isolates recovered from urinary tract infections among female outpatients
Source: PLoS One. 2023 May 11;18(5):e0285427. doi: 10.1371/journal.pone.0285427 (PMC10174568; doi:10.1371/journal.pone.0285427)
Supplement: S1 Table — (DOCX) [file pone.0285427.s001.docx]

**Table S1. Univariate analysis for risk factors of FQ NS and MDR *E. coli* isolated from urine of US women 2015–2019.**

|  | **Study cohort** | **FQ** | | | | | **MDR** | | | | |
| --- | --- | --- | --- | --- | --- | --- | --- | --- | --- | --- | --- |
|  | **N** | **n FQ-S** | **FQ-S, %** | **n FQ NS** | **FQ NS, %** | ***P*** | **n non-MDR** | **non-MDR, %** | **n MDR** | **MDR, %** | ***P*** |
| **Overall** | 1858 | 1489 | 80.1 | **369** | **19.9** |  | 1799 | 96.8 | **59** | **3.2** |  |
| **Age group, years** |  |  |  |  |  | **< 0.0001** |  |  |  |  | **0.0248** |
| 12–17 | 83 | 78 | 94.0 | 5 | 6.0 |  | 82 | 98.8 | 1 | 1.2 |  |
| 18–24 | 320 | 290 | 90.6 | 30 | 9.4 |  | 317 | 99.1 | 3 | 0.9 |  |
| 25–50 | 724 | 600 | 82.9 | 124 | 17.1 |  | 699 | 96.6 | 25 | 3.5 |  |
| > 50 | 731 | 521 | 71.3 | 210 | 28.7 |  | 701 | 95.9 | 30 | 4.1 |  |
| **Prior AMR-ESBL** |  |  |  |  |  | **< 0.0001** |  |  |  |  | **< 0.0001** |
| ≤ 90 days prior to index | 23 | 6 | 26.1 | 17 | 73.9 |  | 14 | 60.9 | 9 | 39.1 |  |
| 91–360 days prior to index | 22 | 12 | 54.6 | 10 | 45.5 |  | 18 | 81.8 | 4 | 18.2 |  |
| No prior AMR | 1813 | 1471 | 81.1 | 342 | 18.9 |  | 1767 | 97.5 | 46 | 2.5 |  |
| **Prior FQ NS** |  |  |  |  |  | **< 0.0001** |  |  |  |  | **< 0.0001** |
| ≤ 90 days prior to index | 55 | 5 | 9.1 | 50 | 90.9 |  | 43 | 78.2 | 12 | 21.8 |  |
| 91–360 days prior to index | 69 | 25 | 36.2 | 44 | 63.8 |  | 65 | 94.2 | 4 | 5.8 |  |
| No prior AMR | 1734 | 1459 | 84.1 | 275 | 15.9 |  | 1691 | 97.5 | 43 | 2.5 |  |
| **Prior SXT NS** |  |  |  |  |  | **< 0.0001** |  |  |  |  | **< 0.0001** |
| ≤ 90 days prior to index | 70 | 38 | 54.3 | 32 | 45.7 |  | 58 | 82.9 | 12 | 17.1 |  |
| 91–360 days prior to index | 76 | 48 | 63.2 | 28 | 36.8 |  | 73 | 96.1 | 3 | 4.0 |  |
| No prior AMR | 1712 | 1403 | 82.0 | 309 | 18.1 |  | 1668 | 97.4 | 44 | 2.6 |  |
| **Prior NFT NS** |  |  |  |  |  | 0.0326 |  |  |  |  | 0.0787 |
| ≤ 90 days prior to index | 31 | 21 | 67.7 | 10 | 32.3 |  | 28 | 90.3 | 3 | 9.7 |  |
| 91–360 days prior to index | 49 | 34 | 69.4 | 15 | 30.6 |  | 47 | 95.9 | 2 | 4.1 |  |
| No prior AMR | 1778 | 1434 | 80.7 | 344 | 19.4 |  | 1724 | 97.0 | 54 | 3.0 |  |
| **Prior positive urine Cx** |  |  |  |  |  | **< 0.0001** |  |  |  |  | **0.0003** |
| ≤ 90 days prior to index | 202 | 135 | 66.8 | 67 | 33.2 |  | 185 | 91.6 | 17 | 8.4 |  |
| 91–360 days prior to index | 253 | 193 | 76.3 | 60 | 23.7 |  | 247 | 97.6 | 6 | 2.4 |  |
| No prior AMR | 1403 | 1161 | 82.8 | 242 | 17.3 |  | 1367 | 97.4 | 36 | 2.6 |  |
| **Prior negative Urine Cx** |  |  |  |  |  | **0.0005** |  |  |  |  | **< 0.0001** |
| ≤ 90 days prior to index | 81 | 53 | 65.4 | 28 | 34.6 |  | 70 | 86.4 | 11 | 13.6 |  |
| 91–360 days prior to index | 97 | 71 | 73.2 | 26 | 26.8 |  | 92 | 94.9 | 5 | 5.2 |  |
| No prior AMR | 1680 | 1365 | 81.3 | 315 | 18.8 |  | 1637 | 97.4 | 43 | 2.6 |  |
| **Prior oral FQ receipt (any)** |  |  |  |  |  | **< 0.0001** |  |  |  |  | **< 0.0001** |
| ≤ 90 days prior to index | 126 | 58 | 46.0 | 68 | 54.0 |  | 111 | 88.1 | 15 | 11.9 |  |
| 91–360 days prior to index | 215 | 143 | 66.5 | 72 | 33.5 |  | 200 | 93.0 | 15 | 7.0 |  |
| No prior AMR | 1517 | 1288 | 84.9 | 229 | 15.1 |  | 1488 | 98.1 | 29 | 1.9 |  |
| **Prior oral non-FQ receipt (any)** |  |  |  |  |  | **< 0.0001** |  |  |  |  | **< 0.0001** |
| ≤ 90 days prior to index | 457 | 318 | 69.6 | 139 | 30.4 |  | 424 | 92.8 | 33 | 7.2 |  |
| 91–360 days prior to index | 419 | 328 | 78.3 | 91 | 21.7 |  | 406 | 96.9 | 13 | 3.1 |  |
| No prior AMR | 982 | 843 | 85.9 | 139 | 14.2 |  | 969 | 98.7 | 13 | 1.3 |  |
| **Prior oral AB receipt (any)** |  |  |  |  |  | **< 0.0001** |  |  |  |  | **< 0.0001** |
| ≤ 90 days prior to index | 516 | 348 | 67.4 | 168 | 32.6 |  | 479 | 92.8 | 37 | 7.2 |  |
| 91–360 days prior to index | 447 | 353 | 79.0 | 94 | 21.0 |  | 435 | 97.3 | 12 | 2.7 |  |
| No prior AMR | 895 | 788 | 88.0 | 107 | 12.0 |  | 885 | 98.9 | 10 | 1.1 |  |
| **Prior hospital admission** |  |  |  |  |  | **< 0.0001** |  |  |  |  | **0.0005** |
| ≤ 90 days prior to index | 59 | 46 | 78.0 | 13 | 22.0 |  | 55 | 93.2 | 4 | 6.8 |  |
| 91–360 days prior to index | 223 | 147 | 65.9 | 76 | 34.1 |  | 207 | 92.8 | 16 | 7.2 |  |
| No prior AMR | 1576 | 1296 | 82.2 | 280 | 17.8 |  | 1537 | 97.5 | 39 | 2.5 |  |
| **Prior hospital admission with IV or oral AB in admission** |  |  |  |  |  | **< 0.0001** |  |  |  |  | **0.0018** |
| ≤ 90 days prior to index | 5 | 3 | 60.0 | 2 | 40.0 |  | 4 | 80.0 | 1 | 20.0 |  |
| 91–360 days prior to index | 23 | 10 | 43.5 | 13 | 56.5 |  | 19 | 82.6 | 4 | 17.4 |  |
| No prior AMR | 1830 | 1476 | 80.7 | 354 | 19.3 |  | 1776 | 97.1 | 54 | 3.0 |  |
| **Hospital bed size** |  |  |  |  |  | 0.0565 |  |  |  |  | 0.053 |
| ≤ 300 | 253 | 214 | 84.6 | 39 | 15.4 |  | 250 | 98.8 | 3 | 1.2 |  |
| > 300 | 1605 | 1275 | 79.4 | 330 | 20.6 |  | 1549 | 96.5 | 56 | 3.5 |  |
| **Teaching status** |  |  |  |  |  | 0.0803 |  |  |  |  | 0.5017 |
| Non-teaching | 1109 | 874 | 78.8 | 235 | 21.2 |  | 1071 | 96.6 | 38 | 3.4 |  |
| Teaching | 749 | 615 | 82.1 | 134 | 17.9 |  | 728 | 97.2 | 21 | 2.8 |  |
| **Region** |  |  |  |  |  | 0.3450 |  |  |  |  | 0.1771 |
| East Central | 769 | 606 | 78.8 | 163 | 21.2 |  | 749 | 97.4 | 20 | 2.6 |  |
| Middle Atlantic | 469 | 375 | 80.0 | 94 | 20.0 |  | 448 | 95.5 | 21 | 4.5 |  |
| West Central & Pacific | 620 | 508 | 81.9 | 112 | 18.1 |  | 602 | 97.1 | 18 | 2.9 |  |

All analyses were conducted using SAS V9.4 (SAS Institute, Cary, NC, USA).

AB, antibiotic; AMR, antimicrobial resistance; Cx, culture; *E. coli*, Escherichia coli; ESBL, extended-spectrum beta lactamase; FQ, fluoroquinolone; IV, intravenous; MDR, multidrug-resistant; NFT, nitrofurantoin; NS, not susceptible; S, susceptible; SXT, trimethoprim-sulfamethoxazole.
